# HGF/c-Met related activation of β-catenin in hepatoblastoma

**DOI:** 10.1186/1756-9966-30-96

**Published:** 2011-10-12

**Authors:** Rachel Purcell, Margaret Childs, Rudolf Maibach, Carina Miles, Clinton Turner, Arthur Zimmermann, Michael Sullivan

**Affiliations:** 1 Children's Cancer Research Group, University of Otago, Christchurch, Christchurch, New Zealand; 2 Children's Cancer and Leukaemia Group, University of Leicester, Leicester LE1 6TH (UK; 3 SIAK Co-ordinating Center, Effingerstrasse 40, Bern, Switzerland; 4 Department of Pathology, Canterbury Health Laboratories, Christchurch 8140, New Zealand; 5 Institute of Pathology, University of Bern, Murtenstrasse 31, H-3010, Bern, Switzerland

## Abstract

**Background:**

Activation of beta-catenin is a hallmark of hepatoblastoma (HB) and appears to play a crucial role in its pathogenesis. While aberrant accumulation of the beta-catenin is a common event in HB, mutations or deletions in CTNNB1 (beta-catenin gene) do not always account for the high frequency of protein expression. In this study we have investigated alternative activation of beta-catenin by HGF/c-Met signaling in a large cohort of 98 HB patients enrolled in the SIOPEL-3 clinical trial.

**Methods:**

We performed immunohistochemistry, using antibodies to total beta-catenin and tyrosine654-phosphorylated beta-catenin, which is a good surrogate marker of HGF/c-Met activation. CTNNB1 mutation analysis was also carried out on all samples. We also investigated beta-catenin pathway activation in two liver cancer cell lines, HuH-6 and HuH-7.

**Results:**

Aberrant beta-catenin expression was seen in the cytoplasm and/or nucleus of 87% of tumour samples. Our results also revealed a large subset of HB, 83%, with cytoplasmic expression of tyrosine654-phosphorylated beta-catenin and 30% showing additional nuclear accumulation. Sequence analysis revealed mutations in 15% of our cohort. Statistical analysis showed an association between nuclear expression of c-Met-activated beta-catenin and wild type CTNNB1 (P-value = 0.015). Analysis of total beta-catenin and Y654-beta-catenin in response to HGF activation in the cell lines, mirrors that observed in our HB tumour cohort.

**Results:**

We identified a significant subset of hepatoblastoma patients for whom targeting of the c-Met pathway may be a treatment option and also demonstrate distinct mechanisms of beta-catenin activation in HB.

## Introduction

Hepatoblastoma is a rare malignant tumor of the liver that occurs in young infants with a median age at diagnosis of 16 months [1]. Hepatoblastoma accounts for 1% of new cancer diagnoses in childhood and is the most common childhood liver cancer [2]. While most cases of hepatoblastoma (HB) are sporadic and its aetiology is unknown, there is a close association of HB with developmental syndromes such as the Beckwith-Wiedemann Syndrome (BWS) and Familial Adenomatous Polyposis (FAP) [3,4].

Several distinct histological subtypes of hepatoblastoma exist. These include wholly epithelial tumours, with pure fetal and mixed fetal/embryonal histology; tumours with mixed epithelial and mesenchmyal features; and several types of transitional, small and large cell undifferentiated tumours [5]. This heterogeneous tumour spectrum appears to reflect distinct patterns of hepatic embryogenesis, indicating a developmental origin for HB, and such tumour heterogeneity may account for their variation in clinical behaviour [6].

Of several distinct developmentally regulated pathways known to be active in hepatoblastoma, such as IGF2/H19 [7,8], Notch [9], and Wnt/β-catenin [9,10], it is the Wnt/β-catenin pathway that is most closely implicated in its origin [9-15]. A common immunohistochemical finding in HB is the aberrant accumulation of β-catenin protein in the cytoplasm or nucleus [11,12,16]. Several previous studies of sporadic HB have identified mutations or deletions clustered in exon 3 of *CTNNB1*, the gene for β-catenin [11-13,15,17-19].

In the absence of Wnt activation, β-catenin is phosphorylated at specific N-terminal serine and threonine residues by the APC/Axin/GSK3β protein complex resulting in its ubiquitination and subsequent degradation, thus maintaining tight control of β-catenin levels within normal cells [20]. Wnt ligand binding inhibits serine/threonine phosphorylation of β-catenin, leading to its cytoplasmic accumulation. Hypophosphorylated β-catenin binds TCF/LEF transcription factors, translocates to the nucleus and activates the expression of many target genes, including those involved in cell proliferation (e.g. c-myc and cyclin D1), anti-apoptosis (e.g. survivin), invasion (e.g. matrix metalloproteinases) and angiogenesis (e.g. VEGF) [20,21]. The vast majority of missense mutations reported in a variety of human cancers (2381/2394) are within the small GSK3β-binding region of exon 3 of the *CTNNB1 *gene examined in our study (http://www.sanger.ac.uk/genetics/CGP/cosmic) and result in aberrant accumulation of β-catenin in the cell.

Canonical Wnt/β-catenin signaling directly alters gene expression and is a key regulator of cell proliferation, differentiation, and apoptosis during normal liver development, so mutation or deletion within the β-catenin gene suggests a crucial role of this pathway in the origins of embryonal liver tumors [22,23](13-15). When stabilized by mutation or deletion in *CTNNB1*, β-catenin causes pathological gene activation and promotes hepatocyte proliferation [24].

However, a disparity exists, because the very high frequency of aberrant β-catenin protein accumulation seen in these tumors cannot be accounted for by mutation or deletion in the *CTNNB1 *gene alone [25]. While direct activation of β-catenin by *CTNNB1 *mutation is common in many tumours, pathologic activation of β-catenin by HGF/c-Met signaling with associated transformation has also been reported in several tumors and its activation has been previously reported in hepatoblastoma [26]. This Wnt-independent activation of β-catenin was identified involving a separate pool of β-catenin located at the inner surface of the cell membrane in association with c-Met [27].

c-Met is the tyrosine kinase receptor for hepatocyte growth factor (HGF), that upon ligand binding undergoes tyrosine autophosphorylation and in turn triggers the activation of several pathways controlling epithelial-mesenchymal morphogenesis, angiogenesis and cell-cell adhesion [28]. In the liver, the HGF/c-Met pathway has a crucial role the activation of liver cell regeneration following injury or partial hepatectomy, and a similar response is seen following kidney and heart injury suggesting a general role promoting tissue regeneration and repair [29]. Elevated serum levels of HGF have previously been reported in children following resection of hepatoblastoma [30,31].

Upon signaling by HGF, c-Met becomes phosphorylated at tyrosine residues Y1234 and Y1235 and in turn tyrosine phosphorylates β-catenin at residues Y654 and Y670, causing its dissociation from c-Met at the cell membrane. Tyrosine phosphorylated β-catenin is protected from serine/threonine phosphorylation and subsequent proteosomal degradation allowing its accumulation in the nucleus where it acts as a TCF/LEF transcription cofactor. Thus, HGF/c-Met related activation of β-catenin occurs independent of the canonical Wnt/β-catenin pathway [21,27,32].

Under the auspices of the International Society of Paediatric Oncology Liver Tumour strategy group (SIOPEL) we have investigated the status of β-catenin activation in tumours from patients prospectively enrolled in the SIOPEL 3 hepatoblastoma clinical trial [33]. Here we report an analysis of the role of HGF/c-Met related β-catenin activation and *CTNNB1 *mutation activation of β-catenin in a large cohort of 84 patients with hepatoblastoma. This characterisation of β-catenin activation by the c-Met pathway may have clinical relevance because several HGF/c-Met small molecule inhibitors are now in early phase clinical trials.

## Materials and methods

### Patients and SIOPEL HB clinical trials

SIOPEL Liver tumor clinical trials are international, prospective, clinical trials run under the auspices of the SIOP Liver Tumor Strategy Group (SIOPEL). Our cohort comprises patients prospectively enrolled into the SIOPEL 3 clinical trial, a randomised study which opened in March 1998, designed to evaluate the effectiveness of preoperative chemotherapy for standard risk (SR) HB with either cisplatin (CDDP) alone or in combination with doxorubicin (PLADO). A detailed description of the SR patient cohort, its clinical features, staging and outcome has previously been reported [33]. SIOPEL 3 patients with high risk (HR) HB were all treated preoperatively with SUPERPLADO, a three-drug combination of Cisplatin, Doxorubicin and Carboplatin and the results have been reported [34]. All patients were recruited to the SIOPEL 3 clinical trial with appropriate informed consent. This specific study was reviewed and approved by the New Zealand Health Research Council Multi-regional ethics committee (MREC).

### Tumor samples

In this study we have accessed a representative cohort of 84 HB patients with clinical, histologic and survival data available for most samples. Both diagnostic and post-chemotherapy samples were available for fourteen patients bringing the total number of samples analysed to 98. In the case of diagnostic samples there was generally just a single formalin-fixed paraffin-embedded (FFPE) tumor block available containing the entire biopsy material on which the diagnosis was made. For each post-chemotherapy case, the most representative FFPE block was identified by examination of slides stained with haematoxylin and eosin (H+E). From the H+E slides, representative tumor and adjacent normal tissue areas were selected by a pathologist (C.M.) for subsequent tissue array construction.

### Tissue Array Construction

A tissue microarray (TMA) was constructed by depositing a 1 mm core of each tumor or normal tissue into a wax recipient block using the Manual Tissue Arrayer I (Beecher Instruments Inc., Sun Prairie, WI, USA). In cases where tumor heterogeneity was evident, different representative areas of the tumor were sampled for TMA construction. The tissue array block was made in duplicate and 4 μm sections of the TMA blocks were cut for subsequent use in immunohistochemical (IHC) analysis. One TMA section was also stained with H+E for evaluation by pathologists (CM +CT).

### Histologic features of the HB samples

The sample cohort consists of 98 samples from 84 patients comprising 62 diagnostic tumour biopsies and 36 post-surgical specimens (both diagnostic and surgical specimens available in 14 cases). Histologic information was available for 91 samples. The tumours were examined centrally and classified as either wholly epithelial (n = 33) or mixed epithelial and mesenchymal (n = 54). One tumour was diagnosed as hepatocellular carcinoma (fibrolamellar type) and one as a small cell undifferentiated (SCUD). The epithelial component was further subtyped as pure fetal (n = 43), embryonal (n = 3) or mixed fetal and embryonal (n = 41). Two tumors were subtyped as macrotrabecular type. Focal anaplasia was seen in three tumors and cholangioblastic features in two tumors. Thirteen cases of osteoid formation were noted in the histology reports with additional osteoid formation in a post-chemotherapy sample that lacked osteoid in the diagnostic biopsy. Teratoid features were noted in seven samples.

### Clinical characteristics of patients for survival analysis

Clinical information that classified patients into the two well-defined risk groups was available for 71 patients in our cohort. Twenty-seven of these were high-risk and forty-four were standard risk. Of these 71 patients, nine were born with low birth weight. PRETEXT classification revealed that there were two PRETEXT stage 1 patients, twenty-two stage 2, thirty-one stage 3 and sixteen stage 4 patients. Only two patients had serum AFP levels of < 100 at diagnosis, making them high-risk. Eight and seven patients had portal vein and vena cava involvement respectively, and extrahepatic intra-abdominal disease was seen in three patients also making them high-risk cases. Metastatic disease was present at diagnosis in thirteen children. Relapse or progression in five HR cases resulted in the death of four patients. In the standard-risk group there were six relapses leading to a single death from disease.

### Immunohistochemistry

Briefly, 4 μm TMA slides were deparaffinized with xylene and ethanol. Antigen retrieval was performed by pressure cooking for 2 minutes in citrate buffer pH6.0. Endogenous peroxidases were blocked with 0.3% hydrogen peroxide and non-specific binding was blocked with normal goat serum. Slides were incubated overnight at 4°C with primary antibodies: Y1234/5-c-Met at 1:300 dilution, Y654-β-catenin at 1:25 dilution and β-catenin at 1:200 (All from Abcam, Cambridge, UK). The EnVision HRP/DAB detection system (Dako, Glostrup, Denmark) was used to visualise the results. Slides were lightly counterstained with haematoxylin. All antibodies were optimized for use in IHC using breast tumour control tissue and the appropriate positive and negative controls were used.

### Evaluation of Immunostaining

Immunostaining for β-catenin was scored as normal membranous, diffuse or focal cytoplasmic and diffuse or focal nuclear staining. Staining for Y654-β-catenin was scored as negative, cytoplasmic and/or nuclear staining. Staining for Y1234/5-c-Met was scored as positive (cytoplasmic) or negative. Each array duplicate was also stained and the results collated. The staining intensity was noted but not factored, as differing age of donor blocks and variation in fixation methods can impact on staining intensity. The IHC results were analysed in conjunction with two pathologists (CM and CT).

### RNA extraction from tumour and normal tissue

Representative areas of tumour were identified on H+E slides by pathologists and a 1 mm tissue core removed from corresponding areas on paraffin blocks. The RNA was extracted using RecoverALL™ Total Nucleic Acid Isolation kit (Ambion, Austin TX, USA) as per manufacturer's instructions. Normal adjacent tissue was also removed and RNA extracted where it was available in 62 cases.

### *CTNNB1 *mutation detection

Samples with the following quality parameters were analysed for *CTNNB1 *gene mutations: Optical density ratio 260/280 of 1.8 - 2.2 and RNA concentration of > 20 ng/ul using a Nanodrop spectrometer (Thermo Scientific, Wilmington, MA, USA). A 150 bp region of the *CTNNB1 *gene was amplified that includes the β-catenin regulatory region of exon 3 (codons 32-45) using the following primer pair (B-Cat3/B-Cat2): 5' GATTTGATGGAGTTGGACATGG 3' and 5' TCTTCCTCAGGATTGCCTT 3'. Samples were reverse transcribed and amplified using One-Step RT-PCR kit (QIAGEN, Dusseldorf, Germany) on a DNA Engine Thermal Cyclar (BioRad, Hercules, CA, USA). Reverse transcription was at 50°C for 30 minutes followed by first strand synthesis at 95°C for 15 minutes. 35 cycles of 30 seconds each of denaturation at 94°C, annealing at 52°C and extension at 72°C were carried out. Each reaction contained 1 μl RNA template, 2 μl of enzyme mix, 0.6 mMol of forward and reverse primers, 400 μM of each dNTP, 2.5 mM MgCl_2 _in a final reaction volume of 50 μl. RT-PCR products were visualised on a 1.5% agarose gel with ethidium bromide. Amplified RT-PCR products were purified using QIAquick PCR purification kit (QIAGEN) as per manufacturer's instructions. Cycle sequencing was carried out on a GeneAmp^® ^PCR System 9700 thermocycler using ABI Prism Dye Terminator Cycle Sequencing Ready Reaction Kit (Applied Biosystems, Foster City, CA, USA) using 20 ng RT-PCR product. Sequencing products were run on an ABI 373A sequencer (Applied Biosystems) and all mutations were verified by sequencing the sense and anti-sense strands. Mutation analysis was carried out using Variant™ Reporter Software (Applied Biosystems) and showed good quality traces spanning the region of interest.

### Tissue Culture

Human hepatoblastoma cells, Huh-6 (JCRB, Osaka, Japan) were routinely maintained in minimum essential media (MEM) containing 10% FBS and penicillin/streptomycin. The human hepatocellular carcinoma cell line Huh-7 (JCRB) was cultured in Dulbecco's minimum essential media (D-MEM) with 10% FBS and penicillin/streptomycin. The cells were serum starved for 24 hours prior to treatment with recombinant human HGF (Invitrogen, Carlsbad, CA, USA) to a concentration of 50 ng/ml for 30, 60, 90 and 120 minutes.

### Preparation of Nuclear and Cytoplasmic proteins extracts

Nuclear and cytoplasmic protein fractions were isolated from the cell lines at the timepoints indicated with the CelLytic™ NuCLEAR™ Extraction kit (Sigma^®^, Missouri, USA). The lysate protein concentrations were determined by bicinchoninic acid protein assay using BSA as a standard (Pierce, Rockford, IL, USA). Aliquots of the samples were stored at -80°C until use.

### RNA extraction from cell lines

Total RNA was extracted from the HuH-6 and Huh-7 cell lines using the PARIS™ Protein and RNA Isolation kit (Ambion) and *CTNNB1 *mutation detection was carried out as outlined above for the two cell lines.

### Gel Electrophoresis and Western Blotting

Approximately 20 μg of protein sample were run on NuPAGE 4-12% BisTris gels (Invitrogen) with MES-SDS buffer (Invitrogen) using the Xcell SureLock™ Mini-Cell (Invitrogen). The protein marker used was Precision Plus Protein™ Standards (BioRad). The iBlot Gel Transfer Device (Invitrogen) was used for western blotting of proteins. The filters were probed with anti-Y654 β-catenin (Abcam, 1:150) and anti-β-catenin (Abcam, 1:1000). The filters were stripped with a mild stripping buffer containing 1.5% glycine, 0.1% SDS and reprobed after each blot. The immunoblots were incubated for 1 hour with the appropriate secondary antibodies coupled to horseradish peroxidase followed by exposure to ECL plus chemiluminescence reagents (GE Healthcare Biosciences, Piscataway, NJ, USA) and autoradiography. Immunoblotting with anti-TBP for nuclear proteins and anti-β-actin for cytoplasmic extract was used to confirm equal loading.

### Statistical Analysis

Results were analysed with StatView software (Abacus Concepts Inc., USA). Statistical comparisons were made using Pearson's Chi-squared test with Yates' continuity correction data. A *P*-value of < 0.05 was considered statistically significant.

## Results

### Aberrant β-catenin expression in hepatoblastoma

**Figure 1:**
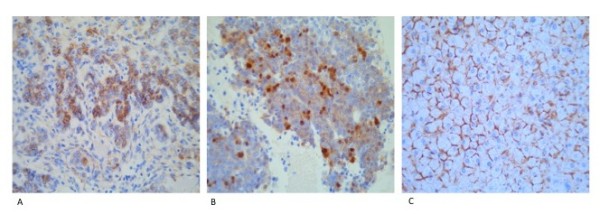
**Immunohistochemical staining of HB using an antibody to β-catenin**. (a) Cytoplasmic staining of β-catenin in hepatoblastoma. (b) Nuclear and cytoplasmic accumulation of β-catenin in hepatoblastoma. (c) Normal staining of the liver cell membrane using an antibody to β-catenin.

We examined total β-catenin protein expression on a HB tissue array using IHC. A total of 87% (85/98) of tumours in our clinical cohort showed aberrant expression of β-catenin in the nucleus and cytoplasm (38/98) or in the cytoplasm alone (47/98) (Figure 1a and 1b). Normal membranous staining alone was observed in seven cases and the remaining six tumours were completely negative for total β-catenin staining. Samples of adjacent normal tissue had a normal membranous β-catenin staining pattern in 46/48 cases available for examination (Figure 1c). The remaining two normal samples showed focal cytoplasmic staining. These results are similar to those published previously in HB studies [18,35,36]. However the frequency of mutations in the *CTNNB1 *gene varies widely in studies of HB, from 13% to 70% [19,37]. To determine whether aberrant β-catenin protein expression is a result of gene mutation, we identified the frequency and type of *CTNNB1 *mutations in our cohort.

### *CTNNB1 *mutation analysis of hepatoblastomas from SIOPEL clinical trial

To identify *CTNNB1 *mutations we extracted total RNA from corresponding tissue cores of hepatoblastoma. A 150 pb region of the β-catenin regulatory region of exon 3 of the *CTNNB1 *gene (codons 32-45) was amplified successfully by RT-PCR in 92 of the samples. Lack of amplification in 6 samples may be due to deletion of exon 3 of *CTNNB1*. We attempted to amplify a region spanning exon 2 to exon 4 in these 6 samples but were unsuccessful. Therefore our estimation of samples containing deletions may be inaccurate. We identified 11 different point mutations in 14 of 98 samples (15%) (Table 1). These are all missense mutations affecting phosphorylation sites in the regulatory region of the gene and have been previously reported [17,38]. The mutations found, resulted in the following changes at the protein level; 32D > N, 32D > Y, 32D > V, 32D > A, 33S > P, 33S > C, 34G > R, 34G > E, 34G > V, 35I > P, 35I > S, 37S > Y. One HB patient (CCRG 64) showed the same sequence variation (missense 32D > V) in both diagnostic and post chemotherapy tumour samples. RNA from adjacent normal tissue was also analysed from 62 cases including nine tumours that harboured mutations. All of these samples displayed wild type *CTNNB1 *showing that the mutations found were somatic variants (results not shown). The frequency of *CTNNB1 *mutations (14/98) and possible deletions (6/98) in our cohort was significantly lower than the frequency of aberrant expression of β-catenin protein and statistical analysis shows no correlation between aberrant β-catenin accumulation and gene mutation/deletion. This prompted us to investigate alternative pathways of β-catenin activation in hepatoblastomas in our patient cohort.

**Table 1:** Histologic type and subtype, β-catenin and Y654 β-catenin IHC and *CTNNB1 *gene status of hepatoblastomas with mutations.

Case Number	Histologic Type	Histologic Subtype	β-catenin	Y654-β-catenin	*CTNNB1 *mutation
CCRG9	Epithelial	Pure fetal	dc	cytoplasmic	32D > Y
CCRG15	Epithelial	Fetal/embryonal	dn	negative	33S > C
CCRG16	Mixed	Fetal/embryonal	dc+dn	cytoplasmic	32D > Y
CCRG48	Epithelial	Pure fetal	dc	cytoplasmic	37S > Y
CCRG61	Epithelial	Pure fetal	dc	cytoplasmic	34G > V
CCRG63	Epithelial	Fetal/embryonal	dn	nuclear	32D > N
CCRG64^a^	Epithelial	Fetal/embryonal	dc+fn	negative	32D > V
CCRG64^b^	Epithelial	Pure fetal	fn	negative	32D > V
CCRG65	Epithelial	Pure fetal	dn	negative	34G > R
CCRG68	Mixed	Fetal/embryonal	dc	cytoplasmic	34G > E
CCRG70	Epithelial	Pure fetal	dc+fn	cytoplasmic	32D > V
CCRG79	Epithelial	Fetal/embryonal	dc+fn	cytoplasmic	32D > N
CCRG82	Mixed	Pure fetal	fc+fn	cytoplasmic	33S > P
CCRG87	Mixed	Pure fetal	dc+dn	cytoplasmic	35I > S
CCRG88	Mixed	Fetal/embryonal	dc+dn	cytoplasmic	32D > V

^a ^Diagnostic specimen from sample CCRG64. ^b ^Post-chemotherapy specimen from sample CCRG64. Abbreviations: dc, diffuse cytoplasmic; dn, diffuse nuclear; fc, focal cytoplasmic; fn, focal nuclear

### High frequency of HGF/c-Met related activation of β-catenin in HB

**Figure 2:**
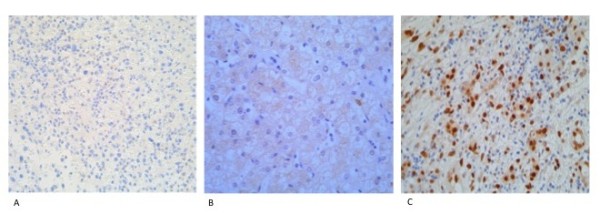
**Immunohistochemical staining of HB using an antibody to Y654-β-catenin**. (a) Hepatoblastoma negative for staining with an antibody to Y654- β-catenin. (b) Diffuse cytoplasmic staining of Y654- β-catenin. (c) Nuclear and cytoplasmic staining of Y654- β-catenin in hepatoblastoma.

To investigate the possibility of Wnt-independent activation of β-catenin, we analysed our tumour cohort for possible HGF/c-Met related tyrosine phosphorylation of β-catenin. We stained the hepatoblastoma tissue array using an antibody recognising tyrosine 654-phosphorylated β-catenin (Y654-β-catenin). This identified positive staining in the cytoplasm of 82/98 (83%) tumours with an additional 27 (28%) showing nuclear accumulation of Y654-β-catenin. In 78 hepatoblastoma with wild type *CTNNB1*, 26 (33%) showed nuclear expression of Y654-β-catenin, 44 (56%) showed cytoplasmic staining with only 7 (9%) negative for staining. In contrast, IHC analysis of 20 hepatoblastoma with *CTNNB1 *mutations or possible deletions showed 5 (25%) were completely negative for Y654-β-catenin (Figure 2a), 14 (70%) had cytoplasmic staining alone (Figure 2b), and only one of 20 (5%) had nuclear expression in addition to cytoplasmic staining (Figure 2c).

Statistical analysis shows a significant correlation between nuclear accumulation of tyrosine-phosphorylated β-catenin and HB tumours with wild-type *CTNNB1 *(*P*-value = 0.015).

**Figure 3:**
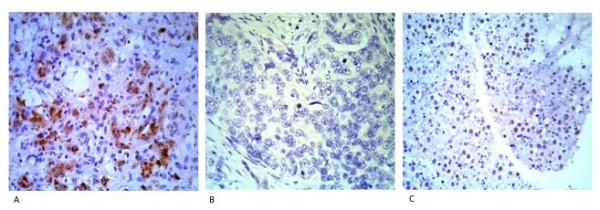
**Immunohistochemical staining of HB using an antibody to Y1234/5-c-Met**. (a) Hepatoblastoma positive for staining with an antibody to Y1234/5-c-Met. (b) Negative staining of Y1234/5-c-Met. (c) Nuclear staining of Y1234/5-c-Met seen in a single case of hepatoblastoma.

To verify that tyrosine phosphorylation of β-catenin is specifically due to activation of the HGF/c-Met pathway we examined the expression of tyrosine 1234 and 1235-phosphorylated c-Met. These tyrosine residues become auto-phosphorylated specifically in response to HGF ligand binding. Eighty-one tumour samples (82%) were positive for Y1234/5-c-Met staining (Figure 3a) and the remaining 17 samples were negative (Figure 3b). A single tumour sample showed a distinct nuclear staining pattern with the antibody to Y1234/5-c-Met (Figure 3c). Statistical analysis showed a 70% correlation between Y1234/5-c-Met and Y654-β-catenin expression (r = 0.7). No correlations between staining patterns and histologic subtypes were found with any of the antibodies used.

### Cell line expression of total β-catenin and Y654-β-catenin in response to HGF activation mirrors that of HB tumours

**Figure 4:**
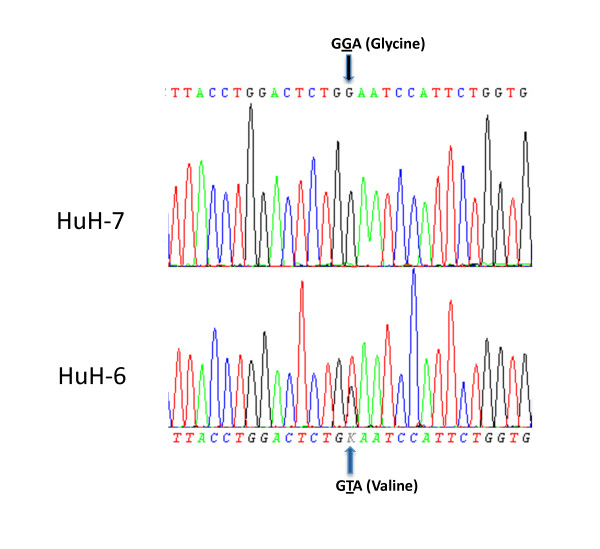
**Direct sequence analysis of exon 3 of β-catenin in HuH-7 and HuH-6 cell lines**. HuH-6 carries a G T transversion, resulting in a glycine to valine amino acid change in codon 34. HuH-7 displays wildtype β-catenin.

To corroborate our immunohistochemistry findings on tissue array, we analysed *in vitro *total β-catenin and Y654-β-catenin protein expression in response to exposure to HGF in two liver tumour cell lines, one with and one without mutation in *CTNNB1 *(Huh-6 and Huh-7 respectively). To determine their *CTNNB1 *status, the Huh-6 and Huh-7 cell lines were analysed for *CTNNB1 *mutations in exon 3 using RT-PCR and sequencing as outlined above. The hepatoblastoma cell line, Huh-6, carried a missense mutation of G34G > V, a known variant of *CTNNB1 *while the hepatocellular carcinoma cell line, Huh-7, was wild type *CTNNB1 *(Figure 4).

These cell lines were then routinely cultured and serum starved for 24 hours prior to treatment with HGF at various timepoints. Total β-catenin expression was assessed by immunoblot of the nuclear and cytoplasmic fractions. As expected the Huh-6 cell line bearing a *CTNNB1 *mutation expressed β-catenin in both nucleus and cytoplasm even in untreated cells (T0) cells due its activating mutation. On exposure to HGF, nuclear and cytoplasmic levels of total β-catenin increased through each timepoint peaking at 90 minutes (Results not shown). In contrast, total β-catenin in the wild type Huh-7 cell line was almost undetectable in the nuclei, and the level seen in the cytoplasm is noticeably lower than that of HuH-6 cells. Upon exposure to HGF, total β-catenin increased in the cytoplasm and was also detected in the nuclei of HuH-7 cells.

**Figure 5:**
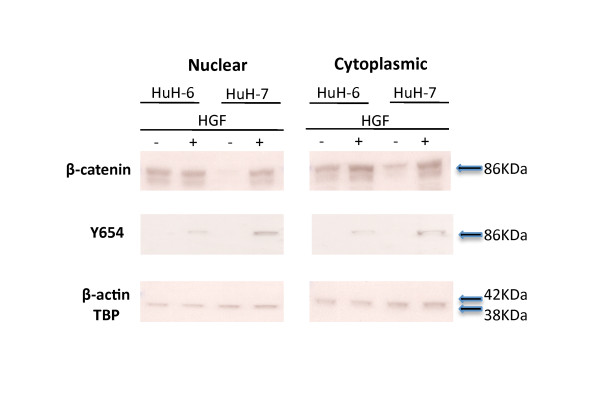
**Immunoblotting of nuclear and cytoplasmic fractions extracted from HuH-6 and HuH-7 cell lines before and after HGF treatment**. Antibodies to β-catenin and Y654- β-catenin were used to probe the blots. Anti-TBP and anti- β-actin were used to ensure equal loading.

Analysis of immunoblots using the Y654-β-catenin allowed us to determine how much of the observed nuclear β-catenin expression may be due to activation by HGF/c-Met rather than an activating *CTNNB1 *mutation. No Y654-β-catenin was seen in any untreated cell fraction, in either the wild type or mutant cell lines. However, upon treatment with HGF the wild type Huh-7 cell line showed significantly more β-catenin expression in the nuclei and cytoplasm compared to Huh-6 (Figure 5).

## Discussion

The accumulation of β-catenin appears to be a crucial event in the tumorigenesis of hepatoblastoma. And although β-catenin gene mutations have been widely reported in hepatoblastoma, a disparity exists between the reported frequency of aberrant β-catenin protein accumulation and mutations in the *CTNNB1 *gene (Table 2).

**Table 2:** Review of previous β-catenin studies in hepatoblastoma

Sample number	Mutation frequency	Deletion frequency	Protein accumulation	References
21	19%	0%	67%	Curia *et al *2008 [36]
17	24%	35%	100%	Yamaoka *et al *2006 [14]
27	33%	37%	-	Taniguchi *et al *2002 [15]
16	31%	44%	-	Udatsu *et al *2001 [19]
68	16%	51%	100%	Takayasu *et al *2001 [18]
30	13%	0%	97%	Park *et al *2001 [37]
18	33%	34%	100%	Wei *et al *2000 [13]
52	25%	15%	-	Koch *et al *1999 [12]

Aberrations in the *CTNNB1 *gene have been reported in up to 75% of HB, with mutation frequencies ranging from 13 - 33% and deletions frequencies of 0 - 51% [12,13,18,19,38]. Our study, in common with several others, has shown a lower frequency of mutations (14%) but a high level of β-catenin protein accumulation (87%) in our sample group [25,36,37]. No deletions in exon 3 of *CTNNB1 *were detected in our sample group, but this may be an under-estimation as we were unable to amplify the gene fragment in 6% of our tumours. The lack of amplification in these samples may be due to RNA fragmentation caused by the formalin-fixation process or may have a true deletion. To err on the side of caution we designated these samples as having possible deletions. Our results serve to corroborate previous studies of β-catenin activation in the pathogenesis of HB in the largest cohort studied to date but the discrepancy in mutation frequencies implies that an alternative activation of β-catenin may occur.

Danilkovitch-Miagkova *et al *showed that c-Met tyrosine phosphorylation of ^®^-catenin has the same effect (same oncogenic transcription) as activation of ^®^-catenin through the Wnt pathway and further studies have implicated c-Met activation of ^®^-catenin in cancer pathogenesis [29,32,39]. More recently, Cieply *et al *investigated hepatocellular (HCC) tumour characteristics occurring in the presence or absence of mutations in *CTNNB1*. The authors found that the fibrolamellar (FL) tumours had the highest tyrosine-654-phosphorylated-^®^-catenin (Y654-^®^-catenin) levels in the study and these tumours also lacked mutations in the *CTNNB1 *gene [40].

This prompted us to analyse our samples for c-Met related ^®^-catenin protein activation. We used an antibodies to detect tyrosine-654 phosphorylated ^®^-catenin (Y654-^®^-catenin) and tyrosine-1234 and 1235-c-Met (Y1234/5-c-Met) as surrogate markers for HGF/c-Met activation. Using this method we found that a large proportion of our cohort (79%) showed c-Met related ^®^-catenin protein activation. Statistical analysis of tumour groups with and without mutations shows a significant correlation between wild type β-catenin and nuclear accumulation of Y654-β-catenin. This is in keeping with the findings of Cieply *et al *in hepatocellular carcinoma. To validate our tumour findings, we looked at the effects of HGF treatment on β-catenin and Y654-β-catenin in two liver cancer cell lines, with and without *CTNNB1 *mutations. The results reflected those seen in HB tumours with c-Met activated β-catenin found only in the cell line with wild type *CTNNB1 *following HGF treatment. It must be noted, however, that nuclear Y654 β-catenin was seen in two tumours carrying mutations/deletions so an overlap of activation pathways may occur. Furthermore thirteen tumours harbouring mutations/deletions also showed Y654 β-catenin expression in the cytoplasm. Further studies must be carried out to ascertain the effect of mutated β-catenin on the nuclear accumulation of the c-Met related β-catenin pool.

**Figure 6:**
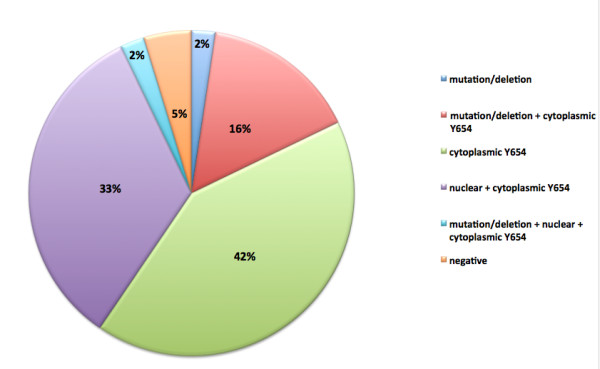
**HB samples with aberrant β-catenin expression showing the breakdown of samples with gene mutations/deletions and Y654-β-catenin protein expression**.

Overall analysis of tumours with aberrant β-catenin expression revealed only a small percentage (5%) that has neither mutations in the *CTNNB1 *gene nor expression of tyrosine654-phosphorylated β-catenin (Figure 6). These tumours may have mutations in other genes such as *AXIN *or *APC *that lead to abnormal β-catenin accumulation or activation through a different pathway. These findings underline that aberrant activation of β-catenin may be critical to the pathogenesis of HB but the means of this activation may not be as important as was previously thought.

Our finding of a large number of tumours (79%) with c-Met activated β-catenin may be relevant to treatment of HB. Although treatment with cisplatin or PLADO followed by resection is highly successful there remains > 15% of HB that suffer from relapse. These relapse patients are often refractive to conventional chemotherapy and have a survival rate of < 20%. The translation of our findings may be important for design of future clinical trials, identifying patients for individual targeted therapy, allowing for fewer side effects or inclusion of c-Met inhibitors in salvage therapy following relapse.

Our findings may also have an application in the treatment of other tumours that display ^®^-catenin activation without associated gene mutation. Somatic mutations in exon 3 of the ^®^-catenin gene have been reported in a variety of cancers (16, 32). However, aberrant accumulation of ^®^-catenin without activating mutations has been reported in cancers such as gastrointestinal carcinoid tumour, ovarian cancer, cutaneous lymphoma, malignant melanoma and pancreatic adenocarcinoma [41-46]. HGF/c-Met activation of ^®^-catenin may account for the discrepancies between gene mutation and protein expression seen in these tumours and this could indicate susceptibility to RTK-targeting agents in the treatment regimen.

## Disclosure of Potential Conflicts of interests

The authors declare that they have no competing interests.

## Authors' contributions

RP carried out the carried out the immunohistochemistry, the molecular genetic studies, the cell culture and protein work and drafted the manuscript. MC participated in study coordination and sample acquisition. RM carried out statistical analysis and contributed to study design. CM and CT analyzed the immunohistochemistry. AZ carried out the initial histologic examination and diagnosis on the tumours. MS conceived of the study, and participated in its design and coordination. All authors read and approved the final manuscript.
